# The function of microbial enzymes in breaking down soil contaminated with pesticides: a review

**DOI:** 10.1007/s00449-024-02978-6

**Published:** 2024-03-08

**Authors:** Xing Kai Chia, Tony Hadibarata, Risky Ayu Kristanti, Muhammad Noor Hazwan Jusoh, Inn Shi Tan, Henry Chee Yew Foo

**Affiliations:** 1 Environmental Engineering Program, Curtin University Malaysia, CDT 250, 98009 Miri, Malaysia; 2 Research Center for Oceanography, National Research and Innovation Agency, Pasir Putih I, Jakarta, 14430 Indonesia; 3 Department of Chemical and Energy Engineering, Curtin University Malaysia, CDT 250, 98009 Miri, Malaysia

**Keywords:** Bioremediation, Microbial enzyme, Pesticide, Contaminated soil, Organochlorine

## Abstract

The use of pesticides and the subsequent accumulation of residues in the soil has become a worldwide problem. Organochlorine (OC) pesticides have spread widely in the environment and caused contamination from past agricultural activities. This article reviews the bioremediation of pesticide compounds in soil using microbial enzymes, including the enzymatic degradation pathway and the recent development of enzyme-mediated bioremediation. Enzyme-mediated bioremediation is divided into phase I and phase II, where the former increases the solubility of pesticide compounds through oxidation–reduction and hydrolysis reactions, while the latter transforms toxic pollutants into less toxic or nontoxic products through conjugation reactions. The identified enzymes that can degrade OC insecticides include dehalogenases, phenol hydroxylase, and laccases. Recent developments to improve enzyme-mediated bioremediation include immobilization, encapsulation, and protein engineering, which ensure its stability, recyclability, handling and storage, and better control of the reaction.

## Introduction

Since the Green Revolution, global food productivity has doubled, and pesticides are among the key drivers [1]. Pesticides have been utilized for decades to prevent diseases transmission by pests such as mosquitoes and fleas, to increase food production by killing insects and pests in farmland [2, 3], and to protect the environment by controlling mold, weed, and algae growth [4, 5]. Crop damage caused by plant diseases, insects and mites, and pest infestations contribute to crop loss, which has a detrimental impact on food security and economies [2, 4–6]. As a result, pesticides are essential for crop cultivation in the agricultural business, particularly for commercially vital crops. Pesticides have been shown to preserve 45% of yearly food output lost due to insect infestation [6]. Over 500 chemicals have been enrolled and used globally as pesticides or pesticide metabolites [5]. In 2018, the worldwide pesticides used in agriculture amounted to 4.15 million tons, with a pesticide application rate of around 2.6 kg/ha [4]. Asia is the leading contributor to the worldwide usage of pesticides, accounting more than fifty percent of the global total. Among the Asia countries, China been reported as the primary consumer of pesticides at 1.77 million tons. [4, 6] Additionally, the United States (0.40 million tons) and Brazil (0.37 million tons) also contribute substantially to the worldwide pesticide market [1, 4, 6].

Despite the fact that pesticides help agricultural crop productivity, the deliberate use of pesticides and the increasing amount of pesticide product manufacturing have had disastrous impacts on the environment. Research done by Eapen et al. revealed that South America and Asia, particularly China, Brazil, Chile, Malaysia, Argentina, and Japan, are the regions with the highest soil pesticide contamination rates [7]. Some of the main causes of the environmental contamination brought on by pesticides include improper field application of pesticides spills, improper cleaning of pesticide storage containers, leaks at pesticide dump sites, and the discharge of industrial effluent containing pesticide from manufacturing facilities [6]. Indeed, pesticide usage and subsequent buildup as residue in soil has become a worldwide concern. Eapen et al. reported that 70% of worldwide croplands had various pesticide residues in the topsoil, with vegetable, fruit, and orchard cropland having the highest pesticide mixture content]. Regardless of the pesticide field application practices, only a limited fraction of pesticides used are effective for their intended uses. It has been estimated that 90% of pesticide residue persists in the adsorbed phase, implying that the majority of pesticides would ultimately end up as residues in numerous environmental compartments including soil, water, and air [7]. As a consequence, pesticide components continue to accumulate in various environmental compartments and when they reach a specific high concentration level, they cause environmental pollution and raise social concerns.

The environmental effects and scope of pesticide contamination are significant and diverse. Despite being created to eradicate pests and increase agricultural yield, pesticides can have unforeseen effects. Pesticides can enter water bodies by runoff from agricultural areas, contaminating the water and harming aquatic habitats. Furthermore, some pesticides' long-term contamination of soil might endanger creatures that are not their intended targets and disturb the ecosystem of the soil. Additionally, pesticides have the ability to bioaccumulate in the food chain and harm human health. The widespread contamination emphasizes how crucial sustainable pesticide management techniques are to reducing environmental damage. Pesticide residues and their metabolites may be transported to various environmental compartments [8], impacting non-targeted creatures such as aquatics, birds, and other organisms in soil and water bodies [7]. Most pesticides' persistent and bioaccumulative properties arouse public health concerns since the active compounds in pesticides might be poisonous and fatal to non-target organisms [3, 5–7]. The toxicity of pesticide residue in diverse environmental compartments may be shown by the instance of pesticide poisoning, which is estimated to cause 1 million fatalities and chronic diseases worldwide each year [5]. Because plants or crops acquire their necessary nutrients from the soil, there is a potential for vegetation to absorb harmful pesticide chemicals from the soil [8] and their presence as residues in human meals [1, 3, 7]. In terms of health hazards, pesticide residues in food and water have been linked to dizziness, breathing problems, neurotoxicity, and chronic poisoning-related disorders such as cancer and mortality rates [3]. Some pesticide compounds have been prohibited from usage due to their toxicity. For instance, organochlorine (OC) pesticides were phased out in the 1970s due to their high environmental persistence and replaced with less persistent organophosphate (OP) compounds [5, 9]. In recent, some OP compounds were also banned from use in Europe and the USA due to their acute neurotoxic nature. Notwithstanding, limiting pesticide usage may significantly lessen soil contamination, but certain extremely persistent chemicals still remain in soils and sediments for a very long period before they are degraded. In other words, despite the fact that OC compounds are no longer allowed to be used as pesticides, their existence in the environment, particularly in soil, is still possible and has been reported [5]. These harmful substances often find their way into the food chain or seep all the way to the water supply [5, 10]. Such a situation calls for effective remediation technologies to remove these contaminants from environmental compartments.

For the cleanup of pesticide-contaminated areas, many approaches have been developed. Existing decontamination methods are classified into physical, chemical, and biological methods [5, 11, 12]. Adsorption and percolator filters are examples of physical treatments. Advanced oxidation, which involves transitory species (OH radicals), is the example of chemical treatment. Biological treatment used a variety of biological systems or microbial populations to bio-transform the toxic compound into a less hazardous or inert one [5, 11]. Among the many pesticide detoxification treatment methods, bioremediation is regarded as a novel and developing method for the cleaning of pesticide-contaminated sites since it provides a sustainable pathway in detoxifying the toxic compounds in soil system [5, 9, 12]. Most importantly, the bioremediation approach is less energy-intensive and eliminates the contaminant without producing byproducts that can cause secondary pollution [5]. In the context of the current trend to use bioremediation for contaminated sites, this review focuses on the bioremediation of pesticide-contaminated soils using microbial enzymes. First, an overview of the status and main challenges of bioremediation in the treatment of pesticide-contaminated soils is presented. Then, bioremediation mediated by microbial enzymes for the treatment of pesticide-contaminated soils is presented including the mechanism and advantages and disadvantages of the treatment methods. The sustainability perspectives of the use of microbial enzymes and the suggestions for future research in the field of microbial enzymatic degradation of pesticides in soil are then presented.

## Types of pesticides

**Fig. 1:**
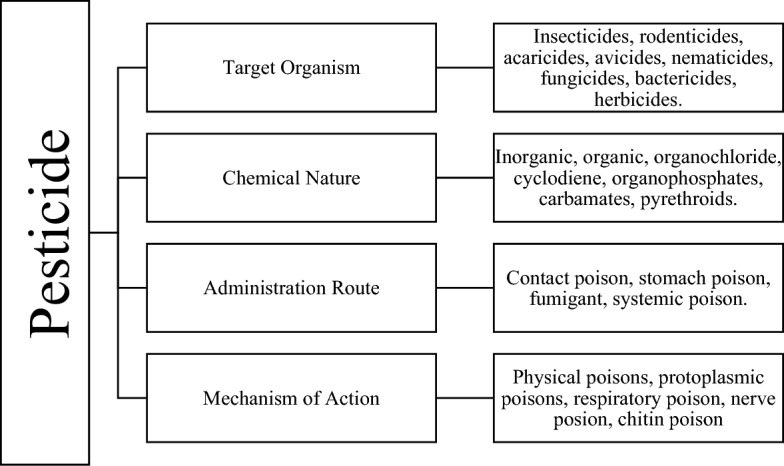
Different ways of pesticide classification

As shown in Fig. 1, pesticides may be categorized according to their target organism, chemical constitution, administration method, and mode of action [3, 4, 13]. Target organism and chemical nature-based classification are the most common pesticide classification method. The former classify pesticide as insecticide, rodenticides, fungicides, etc. The latter classified pesticides as inorganic and organic, where organic pesticide was further classified as organochlorines, organophosphates, carbamates, etc. [4]. Pesticides can be categorized based on their administration or application methods. For example, foliar sprays are applied directly to the leaves of plants, fumigants are gaseous pesticides applied as fumes used to control pests in enclosed spaces or soil, and seed treatments are applied directly to seeds before planting to protect the emerging seedlings [4, 13]. Pesticides can be categorized based on their mode of action, reflecting the specific biochemical or physiological processes they target in pests. Neurotoxins, such as organophosphates (e.g., Malathion) and pyrethroids (e.g., Permethrin), disrupt the nervous system of pests. Insect growth regulators (IGRs), exemplified by Methoprene, interfere with the normal development or reproduction of insects. Mitochondrial inhibitors like Rotenone disrupt cellular respiration in pests by interfering with the electron transport chain in mitochondria [3, 4, 13].

Since the end of World War II, pesticide usage has steadily increased, resulting in the registration of approximately 500 pesticide compounds [5]. Herbicides, fungicides, and insecticides are the three classes of pesticides responsible for more than 95% of the total agricultural pesticides used [4, 14]. Based on the statistic on worldwide pesticide for agricultural use in 2020, the total amount of agricultural pesticides used was 2.66 million metric tons, with agricultural herbicides accounting for almost half of this amount [14]. Some common chemical compounds of herbicides, fungicides, and insecticides are presented in Table 1. The majority of pesticide used todays in agricultural field are synthetic that makes up of organic and inorganic chemical compounds, such as organochlorine, organophosphorus, arsenic, lead, and mercury [4–6]. These constituents more or less toxic to non-target organisms including humans.

**Table 1:** Groups of pesticides based on the basis of target organism and the global use proportion [6, 9]

Groups of pesticide	%	Chemical compounds
Herbicides	55	Phenoxy hormone products, triazines, amides, carbamates, dinitroanilines, urea derivatives, sulfonyl urea, bipyridyls, uracil
Fungicides (include bactericides)	23	Di-thiocarbamates, benzimidazoles, triazoles, diazoles, diazines morpholines
Insecticides	17	Chlorinated HC, organophosphate, carbamates, pyrethroids, botanical and biological products

According to Zhichkina et al. [13] insecticide and herbicide are the most prevalent pesticide residuals in agricultural soil in Samara, Russia [13]. Insecticide compounds discovered in soil include dichloro-diphenyl-trichloroethane (DDT), hydrogen cyanide (HCN), and metaphos. Herbicide compounds discovered in soil include 2,4-dichlorophenoxyacetic acid (2,4-D), dalapon, simazine, atrazine, prometryn, trifluralin, and sodium trichloroacetate (NaTCA). [7, 13, 15, 16] Table 2 provided brief information on the highlighted pesticides, including the characteristics, pesticide group, mode of action, and WHO toxicity rankings. To note, some of them are multifunctional pesticides, for example, HCN and 2,4-D, which are utilized not only as insecticides and herbicides, but also as plant-growth hormone regulators [3, 13, 17]. Different pesticide classes have differing effects on downstream bioremediation. Although enzyme-based methods have potential, not all pesticide classes may be suitable for their use. The efficacy of enzymes for broad-spectrum remediation may be limited by their selectivity towards certain herbicides. Enzyme–substrate interactions and substrate compatibility are important factors. Consequently, the research highlights how crucial it is to evaluate whether enzyme-based tactics are appropriate for every class of pesticide in order to maximize bioremediation results and customize techniques according to the unique properties of the pollutants.

**Table 2:** Information on some common types of pesticide (herbicide and insecticide) residual found in soil [31, 38]

Pesticide	Description	Characteristics	Group	Mode of action	WHO classification
Insecticide					
Dichloro-phenol trichloro-ethane (DDT)	Obsolete and banned insecticide Control insect vectors of disease, such as malaria	Highly persistent in soil Non mobile Low solubility in water Low leachability Low volatility	Organochloride (synthetic)	Non systemic stomach and contact action Sodium channel modulator	II (moderately hazardous)
Hydrogen cyanide (HCN)	Aka hydrogen cyanamide, formonitrile Plant growth regulator to promote bud break	Non-persistence in soil Very mobile High solubility in water Low leachability Highly volatile	Nitrile PGR (synthetic)	Contact Inhibit photosynthesis	Not listed
Metaphos	Aka parathion-methyl Control sucking and chewing insects, such as aphids, armyworms, etc	Non-persistence in soil Moderately mobile Moderate solubility in water Low leachability Low volatility	Organophosphate (Synthetic)	Contact and stomach insecticide Inhibit cholinesterase	Ia (extremely hazardous)
Herbicide					
2,4-Dichloro-phenoxy-acetic acid (2,4-D)	Translocated herbicide, plant growth regulator, and metabolite for use in cereals, grass, and amenity	Non-persistence in soil Mobile High solubility in water High leachability Low volatility	Phenoxy, phenoxy acetic, and Auxin PGR	Selective, systemic, absorbed through roots Increase biosynthesis and ethylene production that cause uncontrolled cell division to damage vascular tissue	II (moderately hazardous)
Dalapon	Used as sodium salt to control annual and perennial grasses such as couch, bluegrass, Quackgrass, etc	Moderately persistent in soil High solubility in water Low volatility	Organochloride or carboxylic acid (synthetic)	Selective, systemic absorbed through leaves and roots Inhibit lipid synthesis	Unlikely to present an acute hazard
Simazine	Soil-acting herbicide to control most germinating annual grasses and broad-leaved weeds	Moderately persistent in soil Moderately mobile Low solubility in water Exist in transition state upon leaching Low volatility	Triazine or chlorotriazine (synthetic)	Selective, systemic, absorbed through roots and foliage and translocated Inhibit photosynthesis	Unlikely to present an acute hazard
Atrazine	Pre- and post- emergence to control broad-leaved weeds and grasses, such as morning glory, Foxtail, etc.	Moderately persistent in soil Moderately mobile Low solubility in water Exist in transition state upon leaching Low volatility	Triazine or chlorotriazine (synthetic)	Selective, systemic action with residual and foliar activity Inhibit photosynthesis	III (slightly hazardous)
Prometryn	Herbicide to control annual grasses and broad-leaved weeds, such as barnyard grass, prairies grass, etc. Moderately persistent in soil and low leachability		Triazine (synthetic)	Selective, systemic, contact, and residual triazine Inhibit photosynthetic electron transport	III (slightly hazardous)
Trifluralin	Pre-emergence soil-incorporated herbicide to control annual grasses and broad-leaved weeds, such as ryegrass, wild oats, pigweed, etc.	Very persistent in soil Non-mobile Low solubility in water Low leachability Moderately volatile	Dinitroaniline (synthetic)	Selective Inhibit mitosis and cell division Inhibit microtubule assembly	Unlikely to present an acute hazard
Sodium trichloro-acetate (NaTCA)	Pre-emergence herbicide to control annual and perennial weeds, such as Quickgrass, Plumegrass, Johnsongrass, etc	Moderately persistent in soil Very mobile High solubility in water High leachability Low volatility	Halogenated aliphatic (synthetic)	Selective, systemic, absorbed by roots and translocated Inhibit lipid synthesis	III (slightly hazardous)

## Environmental fate of pesticides

**Fig. 2:**
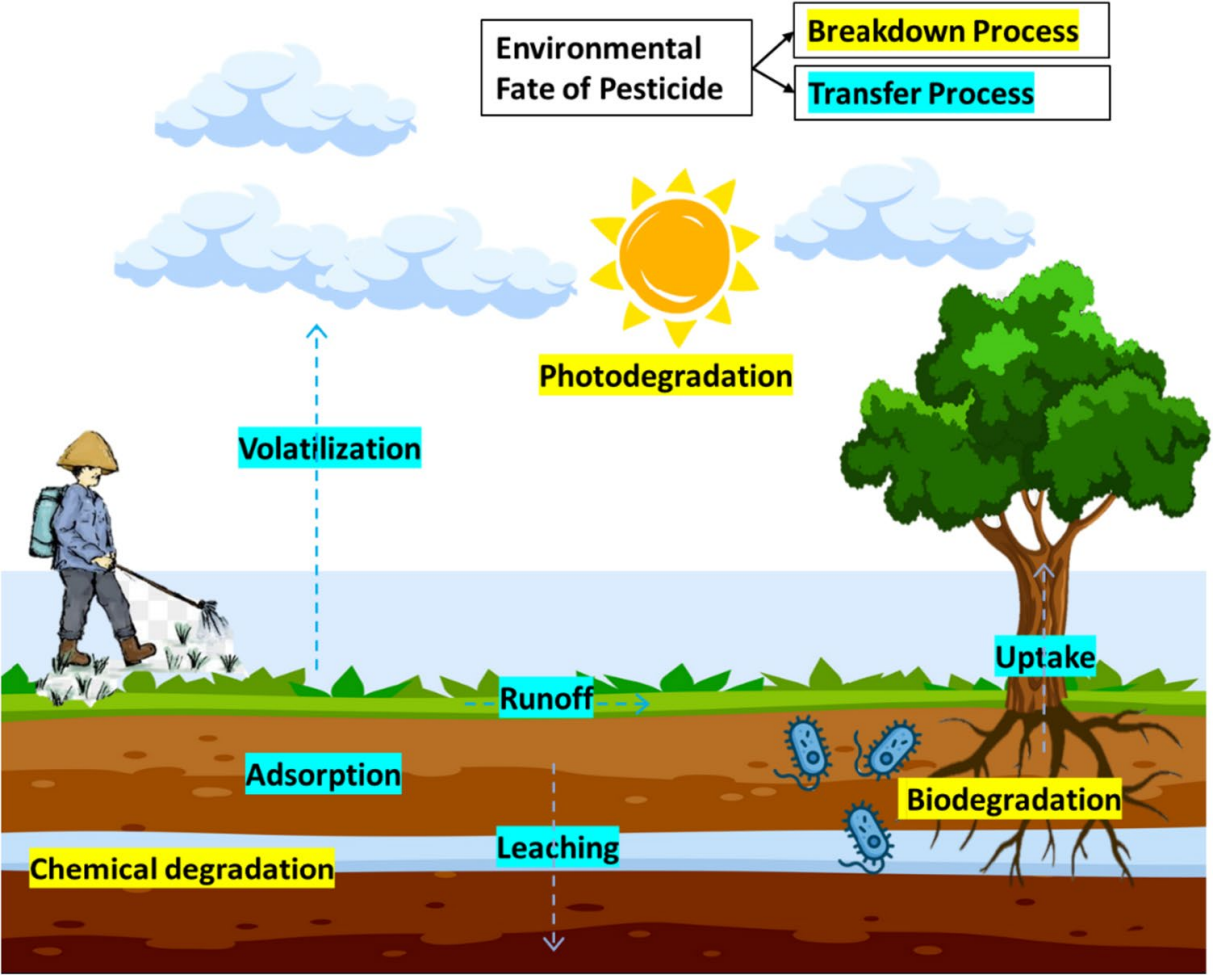
Environmental fate of pesticide [Icon from Flaticon Basic License CC3.0 (Creative Commons)]

**Fig. 3:**
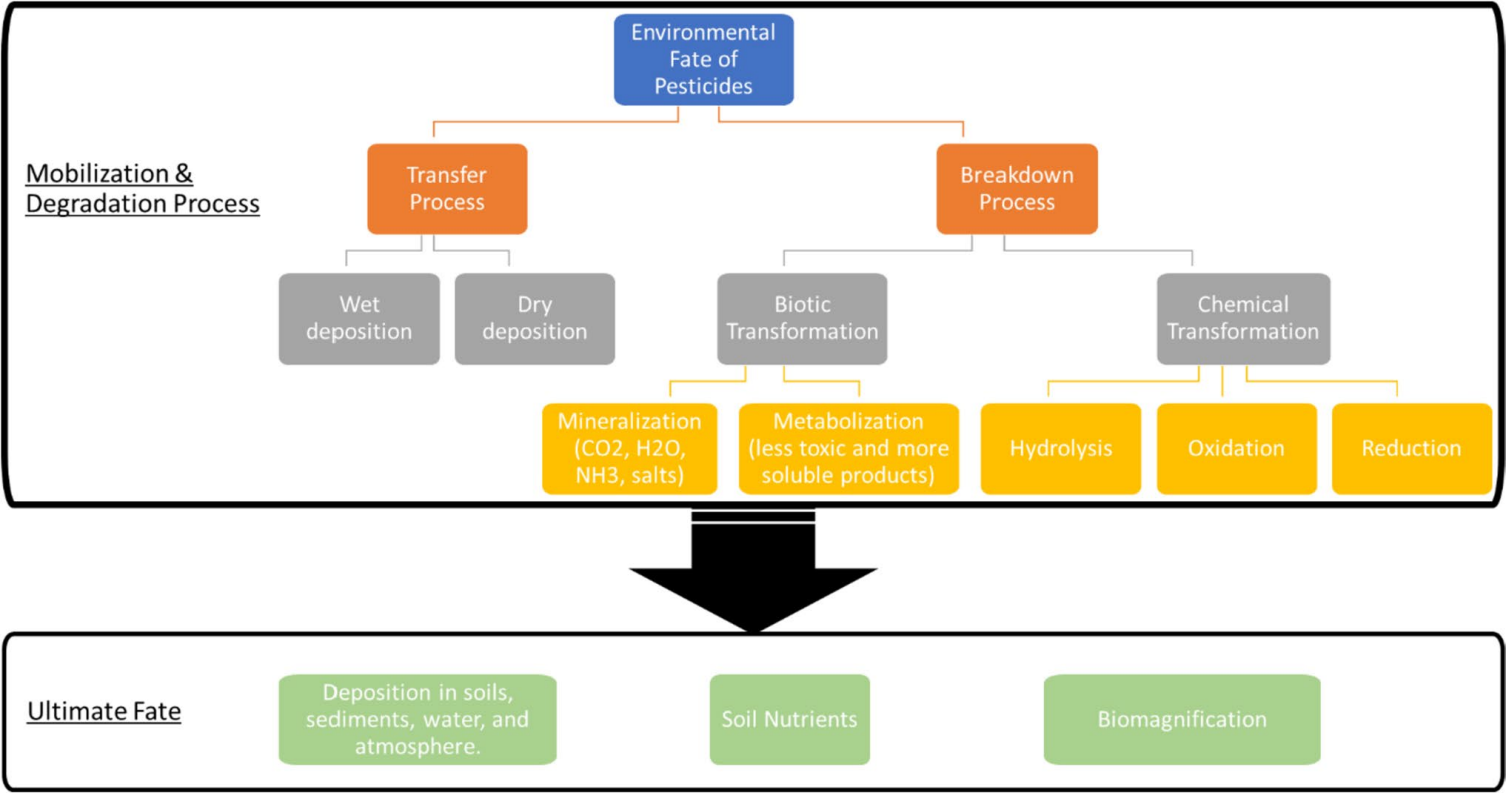
Transfer and breakdown processes of pesticide in soil

Figure 2 illustrates the environmental fate of pesticides, which may be divided into two categories: breakdown and transfer processes. Figure 3 depicts the various mechanisms involved in pesticide breakdown and transfer processes, as well as the ultimate fate of pesticide compounds following the processes. Pesticide transport in soil systems is often driven by erosion and leaching [6–8]. Erosion is the movement of soil particles by wind and water, allowing pesticides to adhere to or desorb from soil particles. Leaching is the gravitational migration of pesticides in the soil via pores and fractures, allowing pesticides to move deeper and reach the groundwater [7, 8]. Furthermore, pesticides are deposited in the atmosphere as a consequence of application drift, wind erosion of treated soil, and post-application vapor loss [8].

Pesticide activity in soil is influenced by a number of complicated dynamic physical, chemical, and biological mechanisms. Once applied, pesticides may enter the atmosphere, adsorb in soil, leach into water, undergo microbiological degradation, and photodegradation [5, 8, 10, 18]. Pesticides adhere to soil particles through adsorption and sorption is often used to describe the attraction of a chemical to soil [8, 13]. Pesticides are highly adsorbed to soil with high clay or organic matter content due to the chemically active and broad surface area that provides more opportunity for pesticide compound sorption. In addition, pesticides are more easily absorbed into dry soil because there is no competition for soil binding sites between water and pesticides. Moreover, pesticides that have been sorbed to soil particles are more likely to persist in the root zone and be accessible for plant uptake as well as microbial or chemical degradation. Furthermore, pesticides that are adsorbed to soil are less likely to volatilize or permeate across the soil [8]. Volatilization is the phase transition of pesticides from solid/liquid to gas, which adds pesticides to the environment through vapor drift [6, 8, 12]. Pesticides are often more volatile in sandy and moist soils, as well as in hot, dry, and windy conditions [8].

Further, the existence of pesticide residue in soil is determined by degradation mechanisms, which include microbial breakdown, chemical interactions, and photodegradation [8, 18]. Microbial degradation is the breakdown of organic matter by microbes such as fungi and bacteria. Moreover, pesticides degrade chemically when they react with water, oxygen, and other substances in the soil. Extremely acidic and alkaline environments often reduce microbial activity while favoring quick chemical reactions that lead to pesticide chemical breakdown within the soil. The breakdown of pesticides by sunlight is referred to as photodegradation [5, 8].

In general, the properties of the compound, such as water solubility, persistency, leachability, mobility, and volatility (vapor pressure), as well as the characteristics of the soil, such as the type of soil, have a significant impact on the pesticide's environmental fate [5, 8, 12, 19]. Table 2 demonstrates the distinct properties of pesticides that attributes to their varying spatial distributions in the environmental compartments. For example, glyphosate, pendimethalin, paraquat, chlorpyrifos, and chlorothalonil were reported to be the most frequently detected pesticide in the topsoil. While dichloropropene, chlorothalonil, metolachors, 2,4-D, and glyphosate were commonly detected below the root zone of soil system, corresponding to their high leaching rate into the ground [7].

Pesticide mobility is one of the important factors that affects their spread throughout the application site [8, 12, 20]. Various pesticides respond differently in different environmental compartments after application. Pesticides, for example, may: (1) remain near the site of deposition by adhering to soil matrices, vegetation, or other surfaces; (2) be attached to soil particles and mobilized with eroded soil by runoff or wind; (3) taken up by plants, or leach to the underground while dissolved in water; and (4) become airborne upon volatilization or erode from soil with wind [8].

Persistency is yet another crucial pesticide characteristic that determines the pesticide's fate in environmental compartments [5, 8]. Half-life measure the persistency of chemical compound; a longer half-life indicates a higher possibility of pesticide migration. Pesticides having a half-life of less than 30 days are often regarded as non-persistent, but those with a half-life of more than 100 days are regarded as persistent pesticides [8]. Highly persistent pesticides are less susceptible to microbiological, chemical, and photodegradation. One of the most persistent pesticides is OC insecticide, which may last in soil for up to a year [9, 11]. Pesticide chemistry, distribution between foliage and soil, as well as environmental factors like pH, temperature, microbial activity, etc., are a few important factors that affect the processes [5, 8].

To recap, once pesticides are released into the environment, they tend to mobilize within the application site and may migrate offsite to reach non-target zones or organisms [7]. This raises concerns about long-term implications of pesticides, since they tend to leach away from the application site and harm the non-target resources and organisms [5–7]. Considering the indispensable use of pesticides and their recalcitrant and possibly biomagnification natures, remediation of pesticide pollutants deem necessary.

## Bioremediation of pesticides (status)

### Bioremediation by microbes

Bioremediation is an innovative and emerging technology for the cleanup of pesticide-polluted sites. Bioremediation is the utilization of natural biological systems (plants and microorganisms) to breakdown contaminants in the environment [5, 12, 21]. Bioremediation is a more economical and environmentally responsible alternative to conventional physical and chemical treatment techniques like oxidation and adsorption. Adsorbent procedures frequently use materials to bind and trap pollutants; these materials need to be changed frequently, which raises the cost of operation. Conversely, oxidation processes frequently necessitate the use of chemicals or energy-intensive techniques, which raises costs and may have an adverse effect on the environment. In contrast, bioremediation uses the innate ability of microorganisms to break down pollutants, providing a low-cost, long-lasting substitute. It is crucial to remember that the efficacy of bioremediation may depend on particular site circumstances, pollutant kinds, and microbial activity, which could provide some restrictions on its application. The total economic and environmental benefits of bioremediation outweigh these drawbacks [5, 12]. The adaptability and versatility of plants and microorganisms have been recognized as having the potential to remove many toxic pollutants created by human activities in a biologically friendly manner [21].

**Fig. 4:**
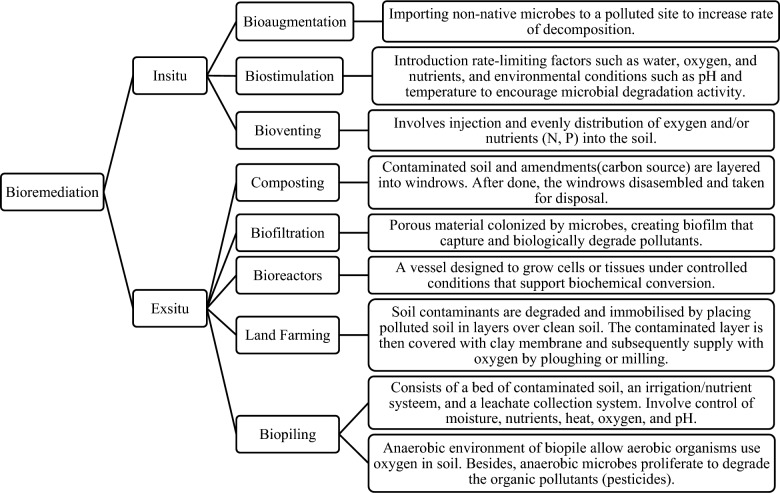
In situ and ex situ bioremediation [9, 18, 52]

Bioremediation treatment using microbes can involve the technology of bioreactors, biologically enhanced soil washing, land farming, composting, etc. [5, 21, 22]. Those treatment technologies can be employed in situ or ex situ [5, 23, 24]. In situ bioremediation involves treatment of contaminated material on the spot, while ex situ bioremediation involves excavation of contaminated material and transport it to elsewhere for treatment under controlled environment [5, 23]. Figure 4 showed the microbial remediation technologies classified under in situ and ex situ bioremediation. Among the most accomplished bioremediation methods are bioaugmentation and bio-stimulation. Bioaugmentation is the introduction of microorganisms into a contaminated environment, while bio-stimulation is the addition of water, oxygen, and nutrients to increase microbial breakdown activity [19].

Bioremediation using microorganisms is becoming more popular for site cleanup. Various microorganisms have been employed to bio-transform pesticides [5, 25]. Bacteria and fungi are the primary organisms engaged in biodegradation [5, 22, 25–27]. Bacterial species discovered to be capable of pesticide degradation often belong to the genera *Flavobacterium*, *Burkholderia*, *Arthrobacter*, *Azotobacter*, and *Pseudomonas* [5, 12, 18, 24, 28, 29]. Once these biotas are released to the soil, they are capable of quickly developing and they breakdown particular pesticide compounds that serve as carbon and energy source to these soil microorganisms, opening the way for the remediation of pesticide-contaminated sites [9, 15, 18, 30]. Furthermore, white rot fungus, *Auricularia auricula*, *Phanerochaete chrysosporium*, and *Dichomitus squalens* are examples of pesticide-degrading fungi [5, 26]. These fungi generate hydrogen peroxide, as well as the extracellular enzyme that are capable of decomposing pesticide compounds. Fungi are responsible for minor structural changes in pesticides that enable them to breakdown into non-toxic compounds and be released into soil, enabling further destruction by natural processes [18, 27].

Microbial degradation includes the processes of oxidation of parent compounds and the subsequent generation of carbon dioxide and water with the release of energy, as well as the production of certain additional byproducts [11, 22, 25]. The process meanwhile supplies carbon and energy for microbial growth and reproduction [15, 22]. Moreover, each degradation stage is mediated by a specific enzyme produced either by the degrading cell or extracellular enzymes [9, 22]. The lack of an adequate enzyme might be the cause of pesticide persistence [5]. Because microorganisms destroy pollutant for the purpose of survival, and organisms accomplish their jobs only under favorable environmental circumstances that meet their demands, certain modifications could be made to encourage the degrading-organisms to degrade the pesticide at a quicker rate in a restricted period of time [15, 22]. To activate the degradation processes of bacteria and fungi, for example, fertilizer or oxygen must be supplied to the pollutant-containing medium. Another example is the exposure to the optimal concentration of contaminant necessary to initiate the metabolic pathways for pollutant digestion by organisms. In general, understanding of the intended microbe's physiology, biochemistry, and genetics is necessary to improve the microbial process and achieve the intended bioremediation [5, 22].

### Development in pesticide bioremediation

The first success of bioremediation was observed in the breakdown of petroleum-derived hydrocarbons by soil microorganisms [21]. Following that, researchers explored to use microbial remediation for other purposes, including environmental decontamination caused by industrial wastes [21, 22, 31]. Currently, microbial remediation is carried out mostly using natural, non-engineered microorganisms that have the potential to metabolize or bio-transform the target contaminant into less toxic compounds [5, 21]. They are isolated from contaminated sources [18, 19].

Despite the advancements in synthetic biology and microbial engineering that have led to the creation of microorganisms with new metabolic pathways or optimization for better fitness in harsh conditions, the use of genetically altered bacteria in the environment remains controversial. The reasons are twofold: first, potential adverse genotypes can be easily mobilized in the environment, which is perceived as a negative attribute of indigenous organisms; second, the unstable nature of inserted genetic material acknowledges that the efficiency of engineered microbes is relied on their tendency to carry the genetic material [19, 21, 22, 27, 32]. As a result, despite the benefits afforded by altered microorganisms, governments such as the United States and Europe restrict the use of genetically modified organisms in the exposing environment [21].

The discovery and isolation of catabolic genes and related enzymes from pesticide-degrading microorganisms is the most recent advancement in xenobiotic pollutants or pesticide biodegradation [12, 22, 33]. A gene encoding an enzyme has been found for various pesticides, providing important insight into the capacity of purified microbial enzyme to breakdown particular pesticide compounds [22, 33]. For example, fungal enzymes such as laccase [18, 34], oxidoreductases [18], and peroxidases [9, 12, 18, 31] been reported to have important applications in pesticide removal. Other bacterial enzymes capable of pesticide degradation include nitro-reductase enzymes from aerobic and anaerobic metabolism bacteria [10, 28, 33] and esterases from *Pseudomonas fluorescens* [25, 34]. Several microorganism enzymes capable of degrading carbofuran, carbaryl, aldicarb, lindane, endosulfan, DDT, and monocrotophos also have been identified [11, 20, 22, 26, 28, 35].

Concerned about the possible negative effects of genetically modified microorganisms, researchers are referring to cell-free synthetic biology technologies, which are distinct from engineered microbes [12, 19, 21]. Cell-free synthetic biology is derived, cell-free catalytic systems with non-replicative properties. It eliminates the environmental constraint brought about by the proliferation of genetically engineered microorganisms and is less likely to be influenced by regulatory regulations [9, 21, 30]. Singh [22] claimed “super strains” in their research study that may degrade the pesticide at a faster rate, attaining the desired bioremediation outcome in a short period of time [22]. Furthermore, Thakur et al. and Jacquet et al. presented the development of recombinant enzymes for environmental cleanup [11, 21]. In general, these research papers advocated enzyme-driven bioremediation from microbial strains rather than whole cell microbes in bioremediation.

Bioremediation using microbial enzymes (enzymatic bioremediation) has been shown to outperform the conventional and bioremediation treatment methods [19, 27]. Table 3 compares the benefits and drawbacks of employing purified enzymes extracted from microorganisms for pesticide breakdown versus using whole microbes. Generally, the use of purified or free enzymes bypasses the limitations associated with the use of whole microorganisms, such as the comparatively slow process of bioremediation, which may take weeks to months for the microbes to accomplish substantive remediation [12, 27, 36]. Furthermore, the performance of microbial remediation is limited to many growth parameters and ideal growth circumstances, such as moisture, pH, temperature, pollutant chemical composition, and redox potential [12, 15, 22].

**Table 3:** Pros and cons of using enzymes to degrade pesticides as compared to the use of whole microbes [29, 34]

Advantage	Disadvantage
Most enzymes are not affected by inhibitors of microbial metabolism	Enzyme production are time consuming and expensive
Enzymes has relatively high physiochemical tolerances than degrading organisms, which can be used under wide range of extreme environmental conditions	Enzymes extracted from cells may be unstable
Can be effective than whole degrading organism in the treatment of low pesticide contamination site	Enzymes may require cofactors which make difficult application
Enzymes remain active in the presence of microbial predators and toxins	Interactions between enzymes and pollutants may be hindered by diffusional constraint
Enzymes preferentially act upon a given substrate rather than the more easily degrade compounds that is prefer by microorganisms	Susceptible to microbial proteases
Enzymes require no uptake mechanism (such as nutrient uptake)	
Enzymes exhibit greater mobility within the soil then microbes	

Enzyme-driven bioremediation, on the other hand, provides enhanced activity for pollutant degradation while producing less waste [12]. Enzymes are biocompatible and biodegradable since they are renewable resources. The capacity of free enzyme to catalyze reactions across a broad temperature and pH range is one of the primary advantages. Furthermore, they provide synthetic pathways that are more step economical, create less waste, and are more energy efficient, making it a more sustainable bioremediation alternative than whole-cell microbial biodegradation. Despite these benefits, the commercial use of free enzyme is limited by its poor operational stability, since enzyme tend to decay quickly in a hostile cell-free environment. Furthermore, the enzyme is sometimes difficult to extract from reaction medium once remediation is complete, resulting in a limited potential of reuse and recovery [12, 37].

Immobilization of enzyme has been suggested to solve the shortcomings of free enzyme application [12, 37]. It refers to the process of physically or chemically attaching the purified enzyme onto or within a support or matrix. To note, immobilized carriers may be made up of biodegradable and non-biodegradable materials. Non-biodegradable carriers may create waste that can potentially re-contaminate the environment; hence biodegradable carriers are preferred. Yu et al. proposed using bacterial cellulose, a biodegradable lining polymer, as the carrier of horseradish peroxidase, which was shown to be capable of degrading chlorophenols [31]. The research found that immobilized enzyme had higher enzymatic activity and operational stability than free enzyme, with the reuse potential of ten cycles. In general, enzyme immobilization sought to increase enzyme activity, stability, and recovery, enabling enzyme to be used in different reaction environment and under challenging circumstances [12]. Table 4 presented the benefits and drawbacks of using free-enzyme and immobilized enzymes for bioremediation.

**Table 4:** Pros and cons of free enzyme and immobilized enzyme for bioremediation

	Free enzyme [29, 33]	Immobilized enzyme [34]
Advantages	Environmentally safe that inhibit the formation of toxic by-products Possibility of simultaneous contaminants bioremediation employing a wide variety of specificity enzymes Enzymatic activity for pollutant degradation happens in a broader environmental condition and adapts to rapidly changing conditions Low activation energy of enzymatic reaction, allowing higher reaction speed and efficient use of energy, which make a more economical and less waste bioremediation There is no need to remove the accumulated biomass from the treated site, as in the case of microbial remediation	Immobilized enzymes are easier to recover and may be reused for detoxification, enhancing their cost-effectiveness Immobilized enzymes exhibited better stability to adverse environmental conditions Enzymes that were immobilized showed greater proteolytic degradation resistance and a longer half-life in soil Easier to handle and store Protein contamination is minimized or avoided The enzyme is less likely to permeate the skin when immobilized to carrier support
Disadvantages	Enzymes tend to lose their reactivity or become inactive after interaction, and unable to reproduce themselves The concentration of enzymes must be controlled and replenished to improve enzyme kinetics Free enzyme is unstable and tend to degrade themselves within a short period of time in a hostile cell-free environment Enzyme activity may be impeded in the absence of a cofactor It is difficult to recover enzymes from reaction medium for reuse Extraction and purification of enzymes is time- and cost- consuming	Not all enzyme that detoxifies can be turned into an immobilized enzyme for bioremediation Carrier support can be expensive

**Fig. 5:**
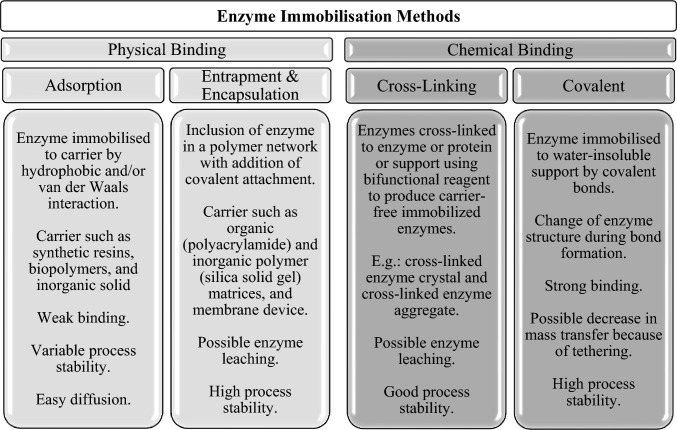
Four types of enzyme immobilization techniques [34, 36]

Figure 5 depicts the several kinds of enzyme immobilization methods, which include physical binding to carrier, entrapment or encapsulation, and cross-linking [19, 28, 31, 37]. Physical binding may be physical, ionic, or covalent, but it is usually inadequate for keeping the enzyme attached to the carrier. Entrapment or encapsulation necessitates the creation of a polymer network that holds the enzyme. Nonetheless, due to the inclusion of a significant amount of non-catalytic ballast, this technique inevitably results in reduced space–time yields and productivities [37]. In this circumstance, cross-linking enzymes with bifunctional regent to form so-called carrier-free immobilized enzymes provided significant benefits [33, 37]. Cross-linked enzyme crystals and cross-linked enzyme aggregates are two examples that provide the benefits of highly concentrated enzyme activity, great stability, and cheap manufacturing costs due to the elimination of carrier expenditures [33, 37].

To conclude, the favorable properties of enzymes lead to their broad use in industries, including the area of decontamination. Recent breakthroughs in biotechnology and protein engineering have enabled the production of enzymes through economically viable processes and their manipulation to display desirable qualities, including substrate specificity, activity, selectivity, stability, and pH optimum. Enzyme-driven remediation is thought to be a recent and emerging trend in the use of environmental decontamination.

## General mechanisms of enzymatic biodegradation

Enzyme-mediated remediation involves the use of enzymes extracted from organisms [18], with this work focused on enzymes isolated from microbial species such as bacteria, fungus, viruses, and algae. Enzymes are biocatalysts or globular proteins that, under favorable circumstances, increase the reaction rate and boost the conversion of substrate into desired products by reducing the activation energy of the process [33, 38].

An enzyme may have one or more catalytically active groups that are incorporated with the active sites via covalent or noncovalent bonds [38]. Enzymes are effective decontamination agents due to their biocatalytic nature and detoxifying properties. They may result in substantial modifications of pollutants’ structural and toxicological properties, as well as their ultimate conversion into benign inorganic end products [34]. For example, oxidoreductases and hydrolases play important roles in the metabolic and catabolic transformation of xenobiotics [39].

Pesticides undergo biotransformation to become less toxic and less persistent. There are two phases of biotransformation: Phase I and Phase II reactions [9, 39, 40]. Phase I of the biotransformation process is critical to reducing the toxicity and persistence of pesticides. Phase I entails a number of chemical reactions that provide the pesticide molecule polar functional groups, including hydroxyl, carboxyl, or epoxide. Usually, these procedures involve reactions involving oxidation, reduction, and hydrolysis. For instance, the addition of oxygen molecules during oxidation might change the structure of the pesticide, increasing its water solubility and promoting more breakdown. In the end, this phase helps to reduce the pesticide's overall toxicity and persistence by making it more susceptible to detoxification in Phase II reactions. Phase II plays a critical role in detoxification. This phase involves conjugation reactions, where the byproducts generated in Phase I undergo further modification through the addition of endogenous molecules. These endogenous molecules, including glutathione, amino acids, phosphate, sulfate, sugars, etc., combine with the Phase I byproducts. This conjugation results in the formation of more water-soluble and less toxic byproducts compared to the original pesticide compound. Phase II reactions enhance the overall biotransformation process, making the pesticide residues more manageable and less harmful to the environment [9, 39–41]. Hodgson presented the comprehensive example of chemical reactions involved in pesticide metabolisms [39]. Generally, most pesticides are bio-transformed via a series of chemical reactions, and the resulting products may become the general metabolic pool.

## Pesticide-metabolizing enzymes and their source

**Fig. 6:**
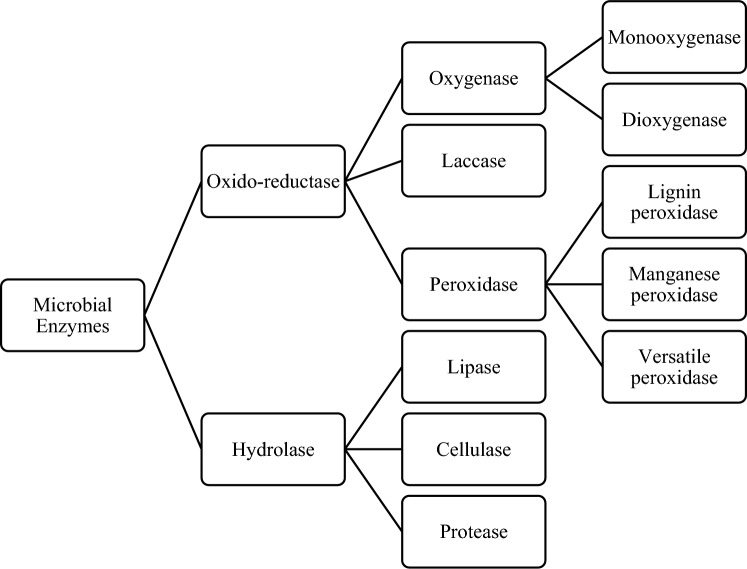
Oxido-reductase and hydrolase group of microbial enzymes [57]

Fungi and bacteria generate extracellular enzymes that accumulate in the soil, leading to the breakdown of pollutants [5, 27, 34]. For instance, laccase, lignin peroxidase, and manganese peroxidase released from fungal mycelium into the surrounding environment [18], have been found to detoxify chlorinated phenolic compounds in soil. Besides, oxidoreductases and hydrolases are the most extensively studied groups of enzymes from different microorganisms (Fig. 6), which mediate degradation processes through oxidation–reduction and hydrolysis reactions, respectively [34, 38]. Oxidation–reduction degradation mechanisms mediated by the oxido-reductase enzyme family, includes oxygenase, laccase, and peroxidase [57]. They were reported to detoxify phenolic or anilinic compounds by catalyzing humification, polymerization, and copolymerization with other substrates [31, 38]. Besides, hydrolytic enzymes lessen the toxicity of pesticides, including OP, OC, and carbamate insecticides, by disrupting their main chemical linkages [16, 24, 26, 57]. Hydrolytic enzymes have wider application because they are readily available, tolerant to water-miscible solvent, and lack cofactor stereoselectivity [38].

As mentioned, the initial reaction involves phase I enzyme that catalyzing an oxidation reaction. Reduction reactions may occur even though relatively uncommon [9, 38]. Cytochrome P450 (CYP) [28], flavin-dependent monooxygenases (FMO) [18, 20], and hydrolases [9, 11, 20, 28, 29, 34] have all been documented to play key roles in the oxidation and reduction of pesticides. CYP undergoes mono-oxygenation processes that metabolize a variety of pesticides, including aldrin through epoxidation, parathion by oxidative desulfurization, and alachlor and atrazine via *N*-dealkylation [30, 39]. FMO only catalyzes oxidation processes when NADPH, a cofactor for CYP-mediated biotransformation, and oxygen as the substrate are present [38, 39]. FMO has been shown in studies to oxidize thioether-containing pesticides such as phosphonate insecticides by oxidative desulfuration with oxon production [39]. Hodgson discusses several Phase I enzymes in detail, including epoxide hydrolases, aldehyde oxidase, prostaglandin synthetase, amidases, and DDT dehydrochlorinase [39].

Following that, the Phase II conjugation reaction pesticide-metabolizing enzymes also explained in detail by Hodgson [39]. Relevant pesticide-metabolizing enzymes include glutathione S-Transferases (GSTs), which are recognized to metabolize OP, DDT, HCH, and organo-thiocyanates; glucuronyl transferases, which metabolize carbamates and OP compounds; and sulfotransferases (SULT), which reported to undergo sulfation and sulphate conjugate hydrolysis in the metabolism of many xenobiotics and producing sulphate esters. Other Phase II enzymes described include methyltransferases, cysteine conjugate beta-lyase, acyl transferases, and phosphate transferases, which metabolize methyl parathion and carbaryl metabolite [39]. It is essential to understand that multiple enzymes are often needed for the breakdown or initiation of a reaction.

Generally, the bioremediation process depends on the enzymatic attack of contaminants that transform them into less toxic or nontoxic products [27, 34, 38]. The next section will describe the biodegradation process of OC and OP pesticides through microbial enzyme-mediated bioremediation. Numerous microbial enzymes, particularly from bacteria and fungi, have been described as efficient pesticide degraders, despite the fact that the majority of the strains were proved to be effective under laboratory settings [38]. Table 5 listed some of the reported pesticide degrading-enzymes including the source of microorganisms and the degradation pathway.

**Table 5:** Example of microbial enzymes capable of degrading xenobiotics, including pesticides

Group of enzymes	Enzymes	Pathway	Source	Target contaminants	Refs.
Oxido-reductases	Laccases	Cleave the aromatic ring and reduce one molecule of oxygen in water, producing free radicals	*Trametes versicolor* *Trametes hirsuta* *Pleurotus ostreatus* *Pycnoporus sanguineus* *Flavodon flavus*	Xenobiotics pesticides; polynitrated aromatic compounds; PAHs; dyes	[40, 41]
	Peroxidases	Catalyze reduction reaction in the presence of peroxides and produce reactive free radicles after oxidation reaction	*Phanerochaete chrysosporium* *T. versicolor* *Coriolopsis polyzona* *P. ostreatus*	PCBs, DDT	[10, 30, 33, 41]
	Oxygenases (Monooxygenase & Dioxygenase)	Catalyze oxidation reaction by integrating oxygen molecules and rendering the substrate more susceptible to subsequent transformation and mineralization	*Bacillus megaterium* *Pseudomonas putida* *Alcaligenes eutrophus*	Aromatic compounds; fatty acids; Toluene; naphthalene; 2,4-D	[15, 16, 24, 42]
Hydrolases	Lipases	Triglycerol broken down into glycerol and fatty acid	*Arthrobacter* sp.; *Pseudomonas* sp.; *Variovorax* sp.; *Bacillus* sub.; *Acinetobacter*; *Klebsiella* sp.; *Rhodopseudomonas* sp.; *Alicyclobaillus* sp., *B. licheniformis*; *B. cereus; Brevibacillus* sp.	Oil spills; OPs insecticides; phenyl-urea herbicide diuron; malathion	[28, 34]
	Carboxyl-esterases	Catalyze hydrolysis reaction of carboxyl ester bond in synthetic pesticides in the presence of water	*Lucilia cuprina*; *B. cereus*; *A. niger*; *Nephotettix cincticeps*	Organophosphorus; Pyrethroid insecticides	[25, 34]
	Phospho-triesterases	Catalyze hydrolysis reaction of phosphotriester in OP compounds	*Flavobacterium* sp. *Pseudomonas diminuta* *Sulfolobus solfataricus Deinococcus radioduran*	Organophosphorus compounds in pesticide	[13, 71, 72]
	Haloalkane dehalogenase	Catalyze hydrolysis reaction that cleave carbon-halogen linkages in the presence of water as cosubstrate	*Sphingobium japonicum* *Sphingobium indicum*	Chlorinated hydrocarbon, halogenated aliphatic compounds (1,2,3-trichloropropane)	[42, 75]

## Enzymatic biodegradation of different targeted pesticides

This chapter discusses enzyme-mediated bioremediation of organochlorine (OC) and organophosphate (OP) pesticides. Table 6 summarizes the documented OC and OP pesticide-metabolizing enzymes derived from microbial sources that will be discussed.

**Table 6:** Example of microbial enzymes reported to be capable of metabolizing OP and OC pesticides

Enzyme	Source microbial(s)	Cofactor	Target Pesticide(s)		Refs.
*Organochlorine (OC) Pesticides*					
LinA (dehydrochlorinase)	*Sphingobium* sp.; *Sphingomonas* sp.	No	Hexachlorocyclohexane (HCH)	[1, 26]	
LinB (hydrolytic dechlorinase)	*Sphingobium* sp.; *Sphingomonas* sp.	No	Hexachlorocyclohexane (HCH)	[1, 26]	
Esd (serine hydrolase)	*Myobacterium* sp.	Flavin and NADH	Endosulfan	[1, 12, 43]	
Ese (monooxygenase)	*Arthrobacter sp.*	Flavin (FMN)	Endosulfan; Endosulfan sulphate	[1, 12, 43]	
CotA (laccase)	*Bacillus subtilis*	No	Endosulfan	[43]	
*Organophosphate (OP) pesticides*					
OpdA (organophosphate hydrolase)	*Agrobacterium radiobacter; Pseudomonas diminuta; Flavobacterium*	Fe^2+^ and Zn^2+^	Phosphotriester insecticides	[1]	
PTE (phosphotriesterase)	*Pseudomonas diminuta*; *Sulfolobus solfataricus; Deinococcus radiodurans*	Zn^2+^	Paraoxon	[15, 17]	
SsoPox (phosphotriesterase-like lactonases)	Sulfolobus solfataricus	Zn^2+^	Paraoxon	[15]	
OPAA (prolidases)	*Alteromonas* sp.	Mn^2+^	Compounds with cleaving P–F, P–O, P–CN, and P–S bonds	[15]	

### Organochlorine (OC)

**Fig. 7:**
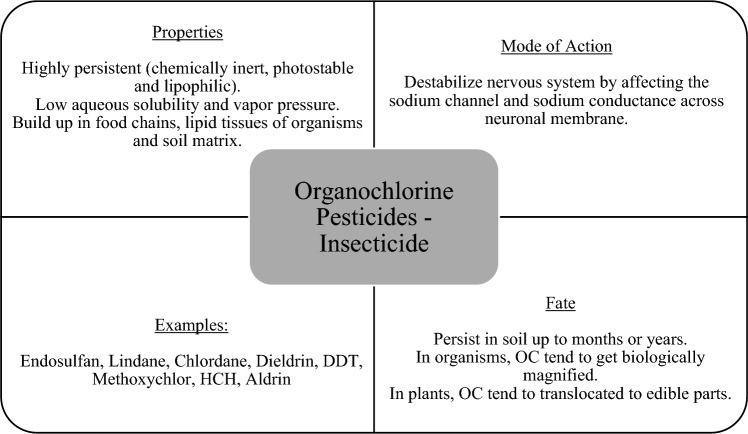
Properties, mode of action, fate, and examples of organochlorine insecticide [9, 12, 26]

Figure 7 provides the basic information about OC pesticides. OC acts as an insecticide in the agricultural field. It is primarily composed of carbon, hydrogen, and chlorine [5]. Dieldrin, HCH, heptachlor, aldrin, endosulfan, dichloro diphenyl trichloroethane (DDT), and methoxychlor, aroclor are common representative compounds in OC pesticides [5, 10, 25]. OC compounds in pesticide are recalcitrant and generally resistant to degradation [5, 25]. As a result, the rate of breakdown is relatively slow and the compounds tend to persist in the environment for a long period of time after application, posing a significant risk of exposure to terrestrial life. Following presented the degradation pathway of OC pesticides by several key microbial enzymes, include dehalogenases on HCH, and phenol hydroxylases and laccases on endosulfan.

#### Dehalogenases: LinA and LinB

Hexachlorocyclohexane (HCH) is an OC insecticide, with a combination of four isomers predominates in the insecticide formulations (alpha-, beta-, delta-, gamma-HCH). Only gamma-HCH (called lindane) is an effective insecticide compound; the other stereoisomers have no insecticide action and are hazardous to non-target organisms. The hazardous nature of these non-insecticidal stereoisomers could be attributed to their chemical composition or biological activity, which may have adverse effects on non-target organisms [10, 42] Several families of bacterial enzymes have been found as capable of degrading the isomer of hexachlorocyclohexane (HCH). *Sphingobium japonicum* UT26 and *Sphingobium indicum* B90A are the most prominent examples [42]. The major pathway of their degradation in soil via aerobic degradation, specifically known as Lin pathway [10]. LinA and LinB are two variants of Lin pathway that reported to be capable of degrading all the four HCH isomers. LinA is a dehydrochlorinase enzyme, and LinB is a hydrolytic dechlorinase enzyme [10].

**Fig. 8:**
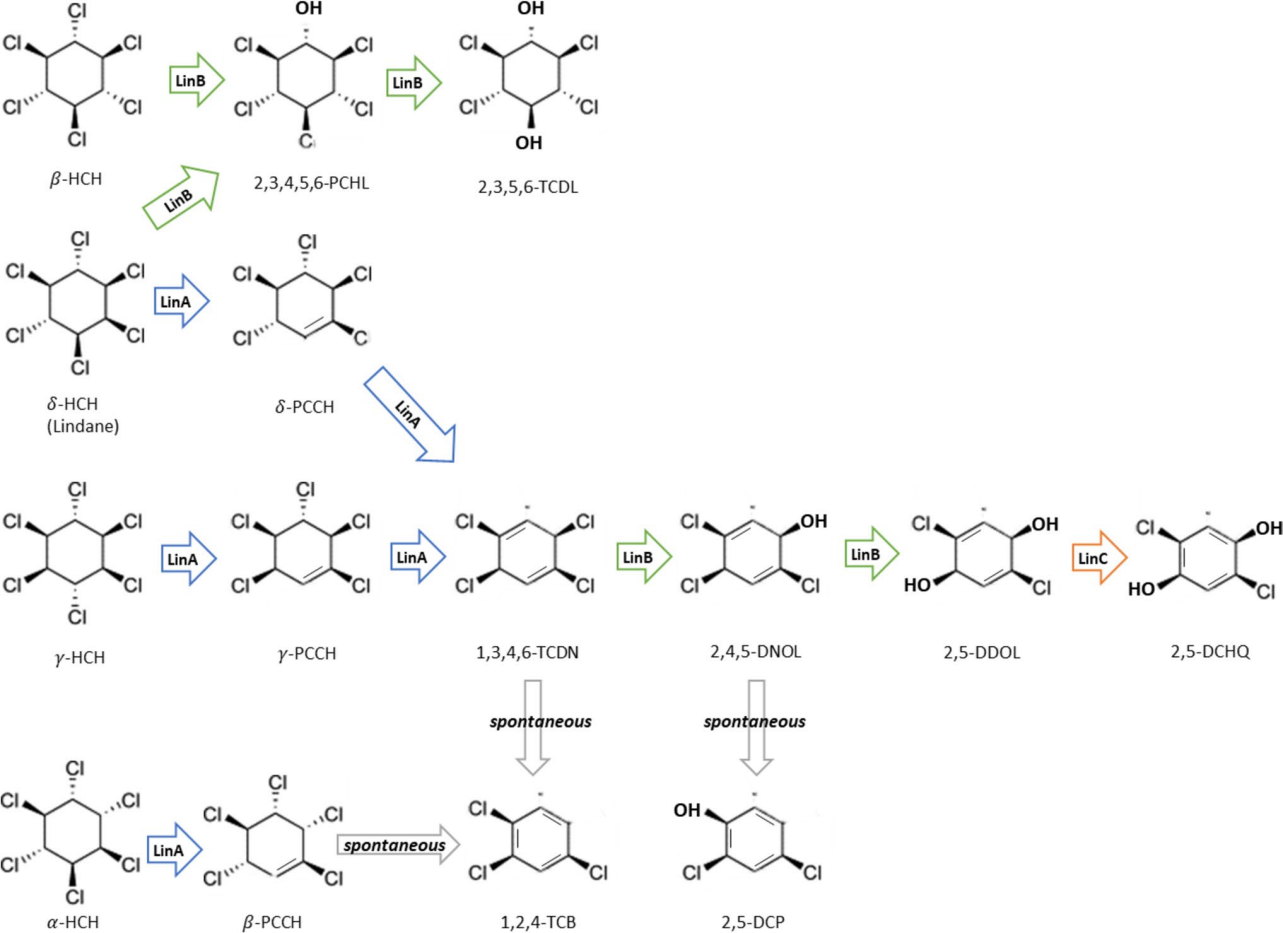
Degradation pathway of HCH isomers by upstream pathway of anaerobic degradation using bacterial enzymes [22, 26, 42]

The degradation process via Lin pathway was extensively studied [10, 42, 43]. Figure 8 demonstrates the degradation pathway. In the case of lindane, the degradation initiated by two LinA from UT26 that catalyzed dehydrochlorinations, producing 1,4-TCDN (1,3,4,6-tetrachloro-1,4-cyclohexadiene) via gamma-PCCH (pentachlorocyclohexene) which is the metabolic intermediates. Then, two LinB catalyzed hydrolytic dechlorination, producing 2,5-DDOL (2,5-dichloro-2,5-cyclohexadiene-1,4-diol) via 2,4,5-DNOL (2,4,5-trichloro2,5-cyclohexadiene-1-ol) [10, 42, 43].

For alpha-HCH, initial degradation initiated by the two variants of dehydrochlorinase or LinA, recognized as LinA1 and LinA2, from strain B90A that enantioselectively transform chiral alpha-HCH to beta-PCCH enantiomers. Then, the beta-PCCH enantiomers degraded through the same pathway as gamma-PCCH. For beta-HCH, the degradation initiated by 2 LinB that catalyzed hydrolytic dechlorinations, producing beta-TDOL (2,3,5,6-tetrachloro-1,4-cyclohexanediol) via beta-PCHL (2,3,4,5,6-pentachlorocyclohexanol). For delta-HCH, two types of dichlorination mechanisms observed. One is the dehydrochlorination of delta-HCH to delta-PCCH by LinA from UT26, while another one is the hydrolytic dichlorination of delta-HCH to tetrachlorocyclohexanediol via PCHL by LinB from B90A [10, 42, 43].

#### Monooxygenases: Ese and Esd

Endosulfan is a cyclodiene OC insecticide that is extensively used for pest control in rice, cotton, cashew, and other agricultural crops [25]. Endosulfan is persistent and abundant in the soil and water environment together with its metabolites due to their poor solubility [12, 44]. The formulation of endosulfan insecticide consists of a 7:3 mixture of alpha isomer and beta isomer [45, 46]. From the microbial degradation point of view, some microbes use endosulfan as their carbon and/or sulfur source [46]. Endosulfan undergoes either oxidation reaction in aerobic condition that form endosulfan sulfate, or hydrolysis reaction in anaerobic condition that form endosulfan diol [5]. Endosulfate is recognized as toxic metabolite having greater persistence than the isomers themselves [25]. Nevertheless, endosulfan sulfate has the higher rate of hydrolysis than endosulfan. Endosulfan sulfate reported to be further transform into endosulfan monoalcohol through oxidation reaction via endosulfan hemisulfate [47].

**Fig. 9:**
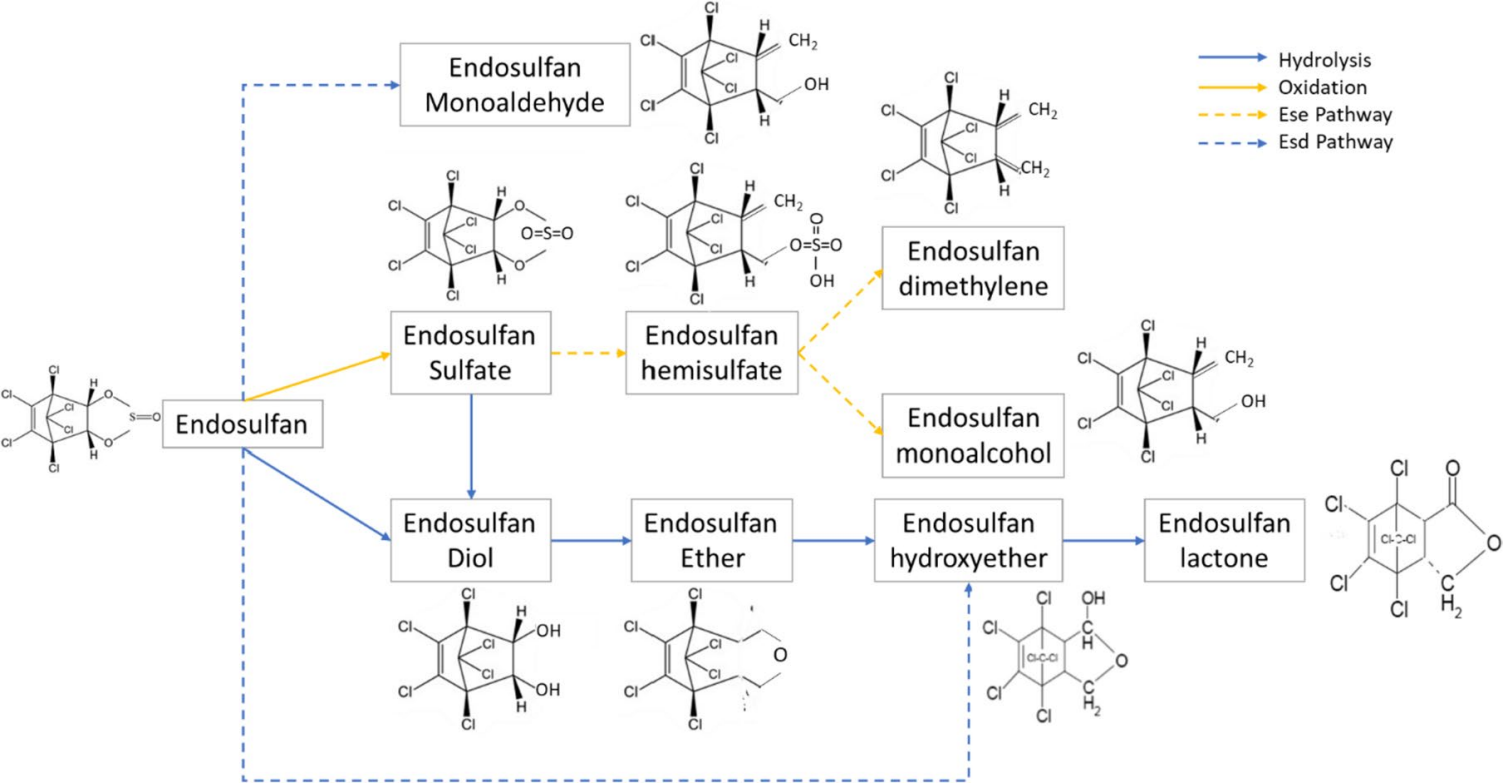
Endosulfan degradation pathways [43, 59]

The biodegradability of endosulfan and its metabolites by enzymes from the family of two-component flavin diffusible monooxygenase (TC-FDM) has been studied in the literature [43, 46, 48]. Ese and Esd are the two members of the TC-FDM family which derived from endosulfan-exposed soil bacteria, capable of degrading the endosulfan and endosulfan sulfate [46]. Figure 9 illustrates the degradation pathway of endosulfan by Ese and Esd enzymes. Esd from *Mycobacterium* sp. has two known routes of degradation. One is to catalyze the oxidation of one of the methylene groups in beta-endosulfan, which results in the production of endosulfan monoaldehyde. The alternative is to catalyze the oxidation of two methylene groups in beta-endosulfan and produce endosulfan hydroxyether [44, 46, 48]. On the other hand, Ese from *Arthrobacter* sp. has been shown to catalyze the oxidation of one methylene group in endosulfan or endosulfan sulfate, producing the unstable sulfur-containing intermediate (endosulfan hemisulfate). The endosulfan hemisulfate is then rapidly desulfurized to yield endosulfan monoalcohol [46, 48].

Furthermore, Singh et al. identified the potential of phenol hydroxylase (1PN0), aka phenol 2-monooxygenase, from *Trichosporon cutaneum* to degrade the alpha-endosulfan (60.36% alpha and 70.73% beta) and endosulfan sulfate (52.08%) [48]. Phenol hydroxylase categorized under class A flavoprotein monooxygenases family having the single dinucleotide binding domain that serve as the coenzyme binding. The coenzyme or cofactor such as flavin adenine dinucleotide (FAD) and nicotinamide adenine dinucleotide (NAD) [48, 49].

#### Laccase

Another bacterial CotA laccase from *Bacillus subtilis* (3ZDW), also proved to be able to degrade alpha-endosulfan, but do not work for endosulfan sulfate. Laccases are known to have a broad substrate range and are capable of oxidizing a variety of aromatic compounds, especially phenolic substrates [49]. Ulčnik et al. found that utilizing bacterial laccase was more efficient than using fungal laccase [50]. Furthermore, the bacterial CotA laccase 3ZDW interacted with endosulfan through strong hydrogen bonds [48]. Endosulfan degradation by the bacterial CotA laccase was reported to have happened without the formation of endosulfan sulphate or any other known metabolites, implying full mineralization of endosulfan [48]. Furthermore, the bacterial laccase CotA from *B. subtilis* has been shown to have a high capacity for lindane degradation [50].

### Organophosphate (OP)

**Fig. 10:**
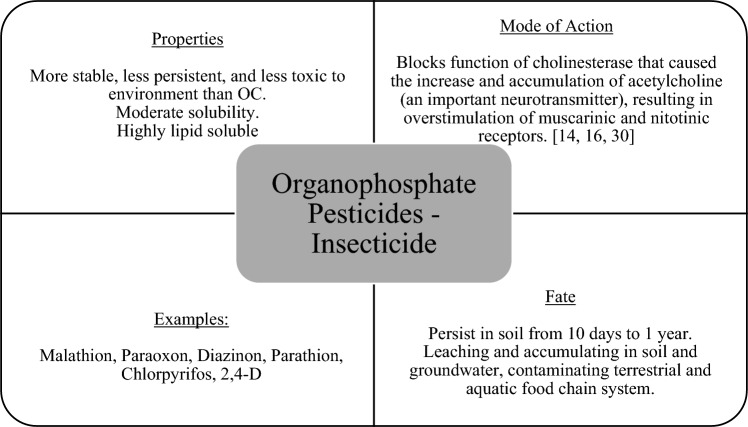
Properties, mode of action, fate, and examples of organophosphate insecticide

OP insecticides have been documented to comprise around 50% of the worldwide insecticide load [51]. The widespread use of OP compounds as an alternative for highly toxic organochlorine compounds in pesticides has resulted in their buildup and contamination of the soil [9, 11]. They are toxic, persistent, and bioaccumulative, contributing to poisoning and environmental harm [11, 21]. Figure 10 presented the properties, mode of action, fate, and representative compound of OP insecticides. It consists of the principal environmental contaminants in soil and water bodies, whose usage has resulted in 3 million intentional poisonings and 300,000 deaths per year worldwide [11]. OP-intoxication is caused by the irreversible binding of OP chemicals to acetylcholinesterase (AChE), that inhibits the enzyme's activity [21]. AChE is a crucial enzyme found in the nervous system of organisms that plays the role of terminating synaptic transmission to avoid continual nerve firings at nerve terminals [52].

While AChE is not capable of degrading OP compounds, the enzyme catalysts found in microbial species have been reported to capable of degrading OP compounds [11, 21, 29]. The most extensively researched are the OP hydrolyzing enzymes, which have emerged as an intriguing approach for decontaminating OP substances. The six primary OP hydrolases are organophosphate hydrolase (OpdA), diisopropylfluorophosphatase (DFPase), phosphotriesterase or OP hydrolases (PTE or OPH), paraoxonase (PON1), organophosphate acid anhydrolase (OPAA), and SsoPox [9, 11, 28, 29].

**Fig. 11:**
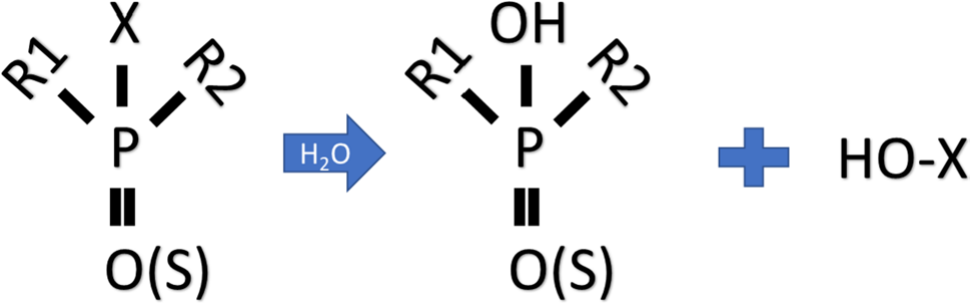
Enzymatic hydrolysis of OP pesticides

Despite each of these six hydrolyzing enzymes having a distinct preference for organophosphorus (OP) compounds, they collectively follow analogous pathways. The enzymatic degradation of OP compounds is shown in Fig. 11. It happens as a result of the compound's phosphorus core being attacked by a pair of divalent metal ions, reactive amino acids, and a water molecule that are present in the enzyme's active site. The OP compound becomes less hazardous when one of its three ester bonds with the main group is broken. The breakdown mechanisms entail oxidation or reduction, followed by hydrolysis. The sequence of breakdown processes leads to the ring cleavage event, which opens up the OP molecule and releases a specific group of compounds to be metabolized further through enzyme catalysis processes. The intermediates produced entered the TCA (tricarboxylic acid) cycle for complete metabolic utilization, releasing CO_2_ and H_2_O as the final products [9].

#### Organophosphate hydrolase (OpdA)

OpdA is among the most efficient OP-degrading enzymes isolated from *Agrobacterium radiobacter,* a saprophytic bacterium found in the agricultural soil [9]. The OpdA enzyme has a barrel structure with a heterobinuclear Fe–Zn metal core, which leads to an increase in specific activity when cobalt is added [9]. The OpdA enzyme has been shown to hydrolyze a broad range of OP pesticides, including chlorpyrifos, diazinon, dichlorvos, dimethoate, malathion, methyl parathion, and ethyl parathion [19, 29]. To illustrate, the hydrolysis of diazinon, an OP insecticide, by the phosphotriesterases OpdA yields diethyl thiophosphoric acid and 2-isopropyl-4 methyl-pyrimidin-6-ol [19].

The phosphotriesterases OpdA are currently used commercially as a free enzyme bio-remediator. LandguardTM, for example, is an OpdA-containing product developed by Orica Ltd Australia and the CSIRO [19, 29]. It is manufactured as a wettable powder to decontaminate the contaminated source. Soil treatment trials with LandguardTM have been found to remove 77% of diazinon within 1 h at an application rate of 100 g/ha [19].

#### Phosphotriesterase (PTE or OPH)

Another enzyme, PTE or OPH, reported to be capable of hydrolyzing OP compounds by breaking P-O and P-S bonds [29, 52]. It has the highest catalytic activity against a variety of OP pesticides and the quickest catalytic rates of any OP-degrading enzyme [21]. PTE was first identified in soil bacteria that hydrolyzed the parathion pesticide [9, 29]. PTE requires divalent metal for its catalytic mechanism, which makes it distinct from other hydrolyzing enzymes [54]. PTE enzyme has a barrel shape with a tertiary protein structure. It is derived from zinc-dependent bacteria which belong to the amidohydrolase superfamily [21]. *Flavobacterium* sp., *Brevundimonas/Pseudomonas diminuta*, *Sulfolobus solfataricus*, and *Deinococcus radiodurans* (aka phosphotriesterase-like lactonases or PLLs) [21, 53] were some of the reported bacterial species.

Past research efforts have been made to enhance functionality of PTE in recognition of its substantial potential as a bioremediation reagent [28]. Immobilization of enzymes to solid supports has been found to increase applicability, with inherent advantages of improved stability and catalytic activity [28]. Raynes et al. observed improved thermal stability when the PTE enzyme was cross-linked to amyloid fibrils synthesized by insulin and crystallin [55]. Furthermore, Karami et al. discovered improved enzymatic and biophysical properties in terms of pH range and temperature when PTE was electrostatically immobilized on Au nanoparticles [56].

**Fig. 12:**
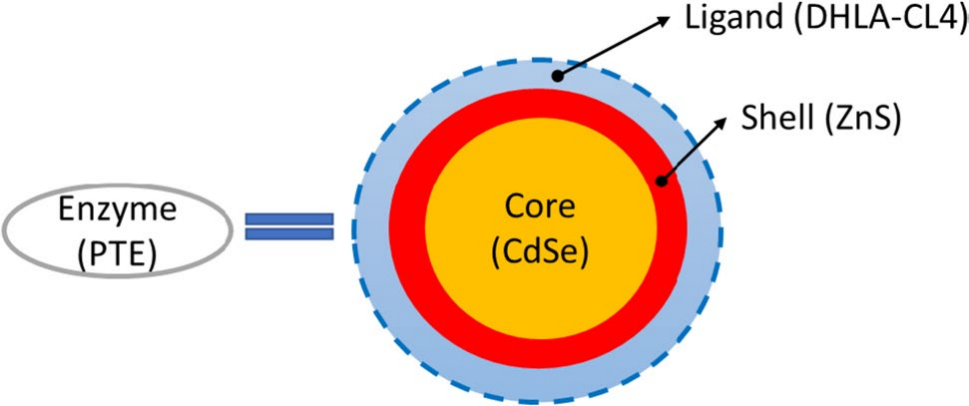
Simple structure of enzyme conjugation to QDs

Additionally, Breger et al. found that PTE conjugation at the interface of semiconductor quantum dots (QDs), a nanoscale material, through metal-affinity coordination enhanced the phosphotriesterase kinetic efficiency by twofold, correlating to higher enzymatic activity [57]. The simplistic structure of enzyme conjugation to QDs is shown in Fig. 12. Generally, the nanoparticle-enhanced catalysis is driven by the PTE-QDs bioconjugation hydration layer that accelerate the enzyme-product dissociation [57]. In terms of the immobilization methods, Breger et al. proposed the immobilization of enzymes to QD using a DNA linker as the conjugate to reduce the biomolecule fouling of the QD surface [58].

#### SsoPox

SsoPox, is another promising field-deployable reagent for bioremediation [21]. It has the folded barrel structure which is similar to the OP hydrolases in the amidohydrolase superfamily [11]. It has been identified as a hyperthermostable enzyme belonging to the phosphotriesterase-like lactonases (PLLs) family and has been found from the archaeon *Sulfolobus solfataricus* [11]. Compared to PTE, SsoPox has lower activity against a number of pesticides, but it is an exceptionally robust enzyme exhibiting activity at temperatures up to 100 °C and in the presence of denaturing agents [21].

Considering the low activity of SsoPox toward phosphotriesterase activity, a structure-based design strategy has been suggested to enhance the active site recognition of SsoPox for a broader range of OP substrates. As an example, Vitola et al. proposed the biocatalytic membrane reactor that was based on covalently immobilizing a triple mutant of the SsoPox on polymeric membranes. The study outcome showed significant paraoxon degradation and long-term stability of the free enzyme [59]. Other PLLs, such as that derived from *Geobacillus kaustophilus* (GkaP), have been found to have capability as OP-degrading enzymes. GkaP cleaves the 6-membered ring structures of lactones, as well as ethyl-paraoxon. [21]

#### Organophosphorus acid anhydrolases (OPAA)

OPAA is bacterial prolidases that cleave P–O, P–CN, P–F, and P–S linkages in OP compounds [21, 29, 60]. OPAA enzymes have a different structure from other bacterial hydrolases, such as PTE and OpdA, implying different OP substrate specificities and activities [21]. Current bio-decontamination formulations for the breakdown of OP compounds come from numerous *Alteromonas* bacteria species [21, 29, 61]. Unfortunately, *Alteromonas* OPAA, like other OP enzymes, has the maximum biological activity at temperatures ranging from 25 to 37 °C, limiting its applicability in field-based applications [21, 60]. *Pyrococcus* sp. was the most recently discovered OPAA [21, 60, 61]. According to Theriot et al., the wild type and mutant prolidases characterized from *Pyrococcus horikoshii* exhibit promising enzymatic capabilities with better thermostability, a larger pH range, and higher metal affinity when compared to *Alteromonas* sp. [60].

## Sustainability prospect of enzymatic biodegradation for pesticide

Remediation of contaminated sites is necessary and beneficial to mitigate the impacts and risks associated with contamination, as well as to restore the ecological functions of the land [62]. However, inappropriate remediation option could introduce impacts of variable scale on the society, environment, and economy [63]. Furthermore, residual impacts may be expected after the implementation of remediation process at the closure stage, especially with regard to the future use of the remediated site [62]. This makes the idea of sustainable remediation pivotal, which incorporates both green and sustainability considerations in the selection and implementation of contaminated site treatment processes, such that overall net benefit is achieved in the aspects of environmental and socioeconomic [62, 64].

Conventional methods of treating contaminated soil, such as soil washing, excavation, land filling, incineration, coagulation-flocculation, chemical oxidation, filtration, and photodegradation are generally time-consuming, expensive, and do not always provide a complete solution, resulting in secondary pollutants [65, 66]. For example, high temperature incineration decomposition offers highly efficient pollutant removal, but it is neither economical nor socially acceptable [65]. Aside from that, typical pollutant removal methods such as membrane filtration and ion exchange have been shown to be incapable of reducing pollutant concentrations to acceptable levels [34]. These chemical and physical decontamination technologies offer the benefit of efficient decontamination but are not sustainable because of their high energy and material requirements as well as the possibility of secondary pollutants generated [65]. Comparing enzyme-based bioremediation to conventional chemical and physical methods reveals that it is a viable, sustainable strategy with substantial economic and environmental benefits. The ability of enzymes to target contaminants with a high degree of selectivity minimizes collateral damage to non-target substances and reduces the production of harmful by-products, making it one of the main advantages for the environment. Enzymatic processes frequently lead to the natural breakdown of contaminants into harmless compounds, in contrast to some chemical treatments that may release toxic residues into the environment. This specificity helps to make the remediation process less harmful to the environment. Furthermore, enzyme-based techniques exhibit inherent compatibility with a wide range of environmental conditions, demonstrating adaptability across multiple ecosystems. Enzyme-based bioremediation can provide solutions that are reasonably priced. Often, renewable and sustainable resources can be used to produce the enzymes.

On the contrary, bioremediation demands fewer resources and energy, and tend to not accumulate hazardous by-products that can cause secondary contamination. It has technical and cost benefits, even though bioremediation might take more time to carry out than conventional methods [65]. Employing microbes for degradation and detoxification of contaminants is now being increasingly employed as the preference technology for site clean-up [33, 66]. In contrast to the conventional approaches, bioremediation more sustainable with less harmful byproducts being produced [21, 33]. In situ bioremediation method is the least expensive polluted site clean-up method because there if no requirement for excavation and transfer of soil [23, 34]. Nevertheless, ex situ bioremediation is feasible for highly contaminated soils with toxic pollutants or when immediate intervention is required. It has been reported that different bioreactors involved in ex situ enzymatic bioremediation provide the best condition for enzymes’ activity, thereby better contaminants removal rate [34].

Bioremediation relies on the enzymatic activity of microorganisms to transform contaminants into less harmful or non-hazardous substances [5]. Numerous enzymes derived from bacteria and fungi have been identified as playing a significant role in the bioremediation of pesticides in soil [33, 34] and discussed in the previous section. It has been claimed that using isolated enzymes has more benefits than using microbial whole cell, including greater specificity, standardizable activity, more convenient in handling and storage, greater mobility because of the smaller size of enzyme, ability to function in the presence of high concentrations of toxic compounds, and biodegradability that prevents persistence and recalcitrance [33, 34, 67]. Significantly, employing enzymes for environmental cleanup facilitates the rapid breakdown of pollutants through various reactions, yielding non-corrosive, non-flammable byproducts that can be safely and easily disposed of [29].

Nevertheless, enzymes are notoriously constrained by the issues of stability, longevity, and reusability issue [33, 60]. The intricately folded structures of the enzyme easily unravel into non-functional amino acid chains or globules once the enzyme is removed from ideal biological conditions of the cell, causing the loss of enzymatic activity [21]. Immobilization has been demonstrated to minimize the decline in enzyme activity, thereby enhancing enzyme stability and longevity [67]. However, not all pesticide-metabolizing enzymes can be immobilized on solid support [19, 27, 33]. Alternative options for enhancement include enzyme encapsulation technology [19, 33, 67]. As an example, PTE-filled outer membrane vesicles (OMVs) with a protein-decorated lipid bilayer provide cargo proteins with protection against environmental nucleases and proteases [21].

Besides, incorporation of enzymes with nanomaterial also been proposed to increase the enzymes’ stability, reduce enzyme susceptibility to mechanical stress, retain the structure of enzymes, and protect the enzyme against proteases [56–58, 70]. Furthermore, recombinant DNA technology and gene engineering have provided more opportunities to produce more efficient enzymes in sufficient quantities [34]. Enzymes may be engineered to enhance their stability and efficiency under certain conditions or with specific substrates [34]. Nonetheless, these green biological technologies may be associated with significant manufacturing costs, as well as a risk of secondary immunological response or environmental pollution [33]. Therefore, it is important that the introduced enzymes to be thoroughly studied on their degradation pathways and intermediates or products produced.

To summarize, sustainable remediation techniques must be less energy intensive, prevent pollutant emissions, and create no disruption or controversy in the surrounding community [62, 64, 70]. Bioremediation using microbial-derived enzyme is a safe, cost-effective, and environmentally friendly method of decontamination [65]. It maximizes natural resource utilization since enzymes are natural sustainable catalysts produced from renewable resources, making them biocompatible and biodegradable [29, 37]. Furthermore, it avoids the usage of chemicals and reduces energy consumption, which are typically required by conventional decontamination methods [3]. Enzymatic bioremediation generates less corrosive byproducts [29]. The greener pathway of pollutant degradation offering by enzyme-mediated decontamination minimize the post-treatment environmental risks, therefore more socially acceptable [33].

**Fig. 13:**
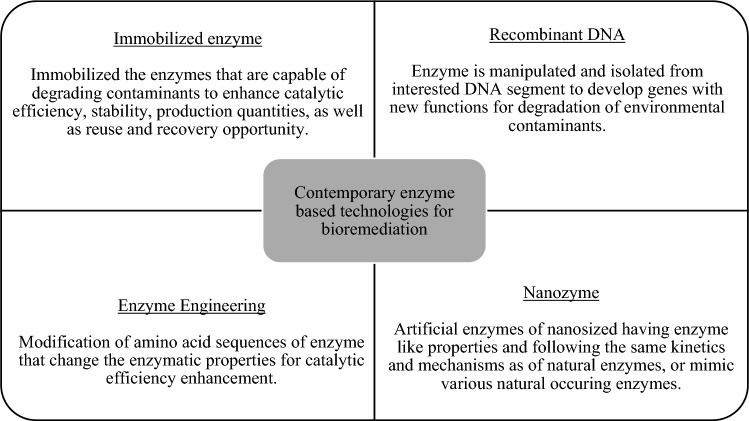
Contemporary enzyme-based technologies for bioremediation

Figure 13 summarized the contemporary enzyme-based technologies for bioremediation. For further improvement, future research can focus on discovering economical nutrient sources for microorganism growth to reduce the production cost of pure enzymes [67]. In addition, mechanisms for enzyme delivery for in situ application should be further explored [23, 33].

## Conclusion

The indispensable use of pesticides, especially herbicides, fungicides, and insecticides, has contributed to their accumulation in various environments that raise the social concern. This article reviews the development and bioremediation pathway of pesticides in soil using microbial enzymes. The conventional chemical and physical methods are commonly used for environmental decontamination, but none of these methods are sustainable. Bioremediation is becoming an indispensable tool to promote a more environmentally friendly and sustainable way of degrading pesticide compounds in soil. Bacteria and fungi are the most common microbial species that can degrade toxic pesticide compounds. They are usually found in contaminated soils. The pesticide acts as a carbon and energy source for these microbial populations, establishing a pathway for the microbial transformation of the pesticide in environmental remediation.

The process of bioremediation is based on enzymatic attack on pollutants to transform them into harmless products. This leads to the trend of developing enzymes as tools for environmental decontamination. Enzyme-mediated bioremediation involves two phases of biotransformation processes. Phase I involves oxidation–reduction and hydrolysis reactions to increase the solubility of pesticide compounds. Phase II consists of conjugation reactions to produce less toxic or nontoxic products. This article focuses on the enzymes released by various microorganisms that are involved in the biodegradation of a wide range of OP and OC insecticides. Several classes of enzymes responsible for the biodegradation of OP and OC compounds in pesticides have been presented. Dehalogenases (LinA and LinB), phenol hydroxylase and laccases are some of the identified OC -degrading enzymes. OpdA, PTE or OPH, DFPase, PON1, SsoPox and OPAA are the most extensively studied OP hydrolyzing enzymes for OP insecticide degradation.

Nevertheless, there are challenges in enzymatic bioremediation related to enzyme stability and high production costs for sufficient quantities of metabolizing enzymes. To address the difficulties in enzymatic bioremediation related to high production costs and enzyme stability, scientists are constantly looking for novel approaches and continuing research projects. Using cutting-edge protein engineering methods to create enzymes with improved stability in a range of environmental circumstances is one interesting direction. Through strategies such as directed evolution and rational design, scientists hope to modify the enzyme's molecular structure to increase its durability and resilience during bioremediation procedures. Additionally, immobilization techniques are being investigated to increase enzyme stability by attaching them to solid supports or matrices. This approach not only enhances enzyme durability but also facilitates their reuse, contributing to cost-effectiveness. Concurrently, research is underway to optimize production processes and reduce the associated costs of producing metabolizing enzymes at a scale. This includes the exploration of alternative expression systems, fermentation strategies, and bioprocessing innovations to streamline production workflows and make enzymatic bioremediation more economically viable. These multifaceted approaches represent a concerted effort to overcome the hurdles posed by enzyme stability and production costs, paving the way for more efficient and sustainable enzymatic bioremediation solutions. In addition, encapsulation technology offers the potential for multi-enzyme compositions to move from simple hydrolysis to multi-enzyme pathways necessary to completely eliminate toxic compounds and produce a much less toxic or non-toxic hydrolysis product. Protein engineering improves the physicochemical properties of natural enzymes so that the enzymes are more tolerant of harsh conditions and exhibit greater efficacy.

In general, considerable efforts are being made to make significant progress in the development of more sustainable enzyme-based bioremediation. Genetic engineering and enzyme engineering become the key focus to reduce the cost of enzyme production, as well as to enhance the activity and stability of enzyme. Nevertheless, more future study should be paid to the application of enzymes under real field conditions since most of the relevant research has been conducted in the laboratory. This is important to understand the factors that can potentially restrict the enzyme activity in order to prove the effectiveness of enzymatic bioremediation. Because environmental systems are inherently complex, researchers face numerous obstacles when conducting enzymatic bioremediation studies in real-world settings. The effectiveness of enzymatic interventions is significantly shaped by varying environmental factors, which makes a thorough approach to study design imperative. Temperature, pH, and moisture content are factors to take into account because they have a significant impact on the stability and functionality of enzymes. The field site's substrate specificity and diversity of contaminants necessitate a careful assessment of the selected enzymes to guarantee their suitability for the particular pollutants found there. Enzyme durability and long-term stability, as well as evaluation of their persistence and reusability, are important factors that must be taken into consideration to guarantee ongoing remediation efforts. Safety and ecological impact considerations go beyond assessing possible.

## Data Availability

Data is available upon request.
