# Correction: The function of microbial enzymes in breaking down soil contaminated with pesticides: a review

**DOI:** 10.1007/s00449-024-03026-z

**Published:** 2024-05-08

**Authors:** Xing Kai Chia, Tony Hadibarata, Risky Ayu Kristanti, Muhammad Noor Hazwan Jusoh, Inn Shi Tan, Henry Chee Yew Foo

**Affiliations:** 1 Environmental Engineering Program, Curtin University Malaysia, CDT 250, 98009 Miri, Malaysia; 2 Research Center for Oceanography, National Research and Innovation Agency, Pasir Putih I, Jakarta, 14430 Indonesia; 3 Department of Chemical and Energy Engineering, Curtin University Malaysia, CDT 250, 98009 Miri, Malaysia

**Correction: Bioprocess and Biosystems Engineering** 10.1007/s00449-024-02978-6

The original version of this article unfortunately contained a mistake, the Acknowledgement is published inadvertently as “This research was supported by Fundamental Research Grant Scheme of Ministry of High Education Malaysia Number 1134. Collaboration from National Research and Innovation Agency is highly appreciated.” and it should be.

“This research was supported by the Ministry of Higher Education, Malaysia, under the Fundamental Research Grant Scheme (FRGS/1/2022/TK06/CURTIN/02/4). Collaboration from National Research and Innovation Agency, Indonesia is highly appreciated.”

The Original Article has been corrected.

